# Patient and tumour characteristics, management, and age-specific survival in women with breast cancer in the East of England

**DOI:** 10.1038/bjc.2011.14

**Published:** 2011-02-15

**Authors:** A M G Ali, D Greenberg, G C Wishart, P Pharoah

**Affiliations:** 1 Department of Public Health and Primary Care, University of Cambridge, Strangeways Research Laboratory, Cambridge, CB1 8RN, UK; 2 Eastern Cancer Registration and Information Centre, Cambridge, UK; 3 Cambridge Breast Unit, Addenbrooke's Hospital, Cambridge University Hospital NHS Foundation Trust, Cambridge, UK; 4 Department of Oncology, University of Cambridge, Cambridge, UK

**Keywords:** breast cancer, relative survival, management, prognostic factors, cancer registry, age

## Abstract

**Background:**

Breast cancer relative survival (BCRS), which compares the observed survival of women with breast cancer with the expected survival of women for the whole population of the same age, time period, and geographical region, tends to be poorer in older women, but the reasons for this are not clear. We examined the influence of patient and tumour characteristics, and treatment on BCRS to see whether these could explain the age-specific effect.

**Methods:**

Data for 14 048 female breast cancer patients diagnosed from 1999 to 2007, aged 50 years or over were obtained from the Eastern Cancer Registration and Information Centre. We estimated relative 5- and 10-year survival for patients in four age groups (50–69, 70–74, 75–79, and 80+ years). We also modelled relative excess mortality (REM) rate using Poisson regression adjusting for patient characteristics and treatment. The REMs derived from these models quantify the extent to which the hazard of death differs from the hazard in the reference category, after taking into account the background risk of death in the general population. We compared the results with those obtained for breast cancer-specific mortality, analysed using multivariate Cox regression.

**Results:**

Median follow-up time was 4.7 years. Relative 5-year survival was 89, 81, 76, and 70% for patients aged 50–69, 70–74, 75–79, and 80+ years, respectively. Corresponding relative 10-year survival was 84, 77, 67, and 66%. Unadjusted REM was 1.93, 2.74, and 3.88 for patients aged 70–74, 75–79, and 80+ years, respectively, (50–69 years as reference). The equivalent hazard ratios from the Cox model were 1.88, 2.45, and 3.81. These were attenuated after adjusting for confounders (REM – 1.49, 1.36, and 1.23; Cox – 1.47, 1.50, and 1.76).

**Conclusion:**

We confirmed poorer BCRS in older women in our region. This was partially explained by known prognostic factors. Further research is needed to determine whether biological differences or suboptimal management can explain the residual excess mortality.

In the United Kingdom, breast cancer accounts for 31% of all cancers in women (Cancer Research UK, 2010). Of these, almost a third occur in women aged ⩾70 years (Cancer screening programme, 2009). Patient characteristics, tumour characteristics, and treatment all influence prognosis in women diagnosed with breast cancer. Tumour characteristics that are major determinants of prognosis are lymph node status, tumour size, histopathological grade, and oestrogen receptor (ER) status (Puglisi *et al*, 1999). Important patient characteristics include age at diagnosis and presence of significant comorbidity (Janssen-Heijnen *et al*, 2005). The relationship between age, patient characteristics, treatment, and prognosis in women with breast cancer is complex. For example, older women tend to present with later-stage disease but are more likely to have suboptimal management, and are more likely to die from comorbid conditions (Diab *et al*, 2000; Eaker *et al*, 2006). Despite this, it is clear that relative survival in older patents is poorer than that of younger patients (Eaker *et al*, 2006; Cancer screening programme, 2009). The reasons for the poorer prognosis in older patients are not clear.

Suboptimal management might also be important. Wishart *et al* (2010) showed that age-specific variation in treatment, particularly in the use of adjuvant hormone and chemotherapy might be attributed to lack of information in older women, in particular nodal status and ER status. The same study also documented that women >70 years of age, who had surgery as part of their treatment, had better overall survival. Several reports have shown that less aggressive patterns of diagnostic activity and care are provided to elderly breast carcinoma patients (Hebert-Croteau *et al*, 1999; Woodard *et al*, 2003), but the impact of these differences on breast cancer survival remains controversial. Gajdos *et al* (2001) found that rates of recurrence were not increased when undertreated women (older than 70 years) were compared with conventionally treated patients, whereas others have found that undertreatment is associated with recurrence and decreased survival (Bouchardy *et al*, 2003).

Variations in care given, and hence prognosis in old patients may be due in part to lack of published guidelines for diagnosis and treatment. This in turn is partly caused by the fact that few randomised controlled trials (RCTs) of breast cancer treatments have included women ⩾70 years old, and most observational studies have been limited by small sample sizes in this age group (Hebert-Croteau *et al*, 1999; Gajdos *et al*, 2001; Bouchardy *et al*, 2003). Recently Schonberg *et al* (2010) have shown that old women have breast cancer characteristics similar to those of younger women, yet receive less aggressive treatment. They suggested that further studies focusing on identifying tumour and patient characteristics are required, which would help target treatments to the oldest women who are most likely to benefit.

The unfavourable breast cancer survival in old women is even more prominent in the UK compared with its counterparts in many other equally developed countries (US, Sweden, Norway, and Australia) (Woods *et al*, 2009; Moller *et al*, 2010). Some studies suggested that a large proportion of differences in survival between the UK and other developed countries can be attributed to differences in stage at diagnosis (Sant *et al*, 2001, 2003).

The aim of this study was to estimate age-specific relative survival in women with breast cancer from the eastern region of England and to explore factors that might explain differences. We also compared the results of the relative survival analyses with the results from an analysis of breast cancer-specific mortality.

## Patients and methods

### Patient data

We used cancer registration data from the Eastern Cancer Registration and Information Centre (ECRIC). All women aged ⩾50 years and diagnosed from 1999 to 2007 with invasive breast cancer (ICD10-site code C50) were eligible for inclusion. During this period, ECRIC covered a population of 2.7 million people in the counties of Bedfordshire, Cambridgeshire, Norfolk, and Suffolk. Cases diagnosed at autopsy or ascertained from death certificate only (DCO) were excluded from the analysis. Eastern Cancer Registration and Information Centre routinely collects data on tumour size, lymph nodes involved, histopathological grade, ER status, mode of detection (screen detected or symptomatic), hospital of diagnosis, and treatment modalities. Patient follow-up is carried out through death certification by the National Health Service Strategic Tracing Service.

Cases in which women were diagnosed with bilateral synchronous breast cancers, data for the most advanced tumour were used in the analysis. In women with metachronous tumours, only the data relating to the first diagnosis were used. Women who had a previous diagnosis of another cancer were excluded. These patients were excluded because survival might be influenced by the previous cancer. This is a standard practice in the population-based survival analysis and has been used previously in many studies (Berrino *et al*, 1998; Capocaccia *et al*, 2003).

The primary sources of registration and treatment data are reports obtained from all pathology laboratories and hospital patient notes from all major NHS hospitals in the region. These are viewed by registry staff, who are either based at the hospital or visit them at least on a monthly basis. Both electronic and paper-based reports are received by the registry, so a high level of completeness of registration is expected. Quality controls included routine plausibility checks on diagnosis, morphology, topography, age, dates, and check on completeness, as well as controls on compatibility of the variables used for staging.

Tumour stage was coded using the TNM classification system for disease stage at the time of diagnosis (Sobin and Wittekind, 1997). The grouped TNM stage in this data included the pathological stage group, augmented by the clinical stage group when the pathological stage was not recorded. The histopathological grade is the degree of differentiation. Cases described as ‘well differentiated’ were assigned as grade 1; ‘moderately differentiated’ as grade 2; ‘poorly differentiated and undifferentiated’ as grade 3, and ‘ungraded (including grade Gx and missing data),’ grade unknown. Volume of treatment at hospital of diagnosis during the study period was categorised as high (465 patients or more throughout the study period) or low (less than 465 patients) throughout the study period. Treatment was classified using indicator variables (yes/no) for surgery, adjuvant chemotherapy, adjuvant radiotherapy, and adjuvant hormonal therapy.

The measure of deprivation used by the UK Association of Cancer Registries in cancer survival analysis is the income domain score of The Index of Multiple Deprivation (IMD, 2004) for each lower level super output area of residence. These scores are grouped into fifths, based on their rankings for the whole of England. The IMD is a standard measure of deprivation at small area level across England. The IMD is based on seven domains – income, employment, health and disability, education and skills, barriers to housing and services, living environment, and crime. Each domain in turn is based on a number of indicators, with some domains also split into subdomains. The full data sets include scores and ranks at small area level for the IMD, domains (and subdomains), individual indicators, and population denominators (The English Indices of Deprivation 2004: Summary (revised), 2010).

For the analyses of breast cancer-specific mortality, a death was assumed to be from breast cancer in which breast cancer was recorded as a cause of death in part 1 of the death certificate.

### Statistical methods

Survival time was measured from the patient's date of diagnosis until death or 30 November 2009, whichever came first. To allow for any delay in death notification, ascertainment of vital status was as of 31 May 2010.

We estimated the relative 5- and 10-year survival rate for patients in four age groups (50–69, 70–74, 75–79, and 80+ years). The relative survival rate (RSR) is an analogue of excess mortality and is a commonly used measure for analysing the survival of cancer patients in population studies, in which cancer-specific mortality might be inadequately ascertained. RSRs adjust all-cause mortality for competing causes of death that would be expected for persons of the same age and sex, time period, and geographic region as the breast cancer patients in the study, without requiring information on the actual cause of death of each patient. Relative survival analysis was performed in Stata using the strs command. The expected survival rates were derived using single year of age and sex-specific death rates from the East of England life tables.

To study differences in survival between different age groups, while adjusting for the confounding factors available in the data set, we modelled relative excess mortality (REM) using Poisson regression (Dickman *et al*, 2004). The REMs derived from these models quantify the extent to which the hazard of death differs from the hazard in the reference category, after taking into account the background risk of death in the general population. We chose women aged 50–69 years as a reference group, as we found little evidence for differences in relative survival within this age group (data not shown). In addition, women aged 50–69 years are eligible for mammography screening under the NHS breast screening programme, and treatment protocols for this age group are reasonably well defined.

The prognostic importance of age, TNM stage, histopathological grade, ER status, mode of detection, deprivation quintile, volume at hospital of diagnosis, and treatment was analysed by both uni- and multivariate-relative survival models. The multivariate analysis was undertaken by taking into consideration all the prognostic factors examined in univariate analysis. Different interactions were investigated for their effect on outcome by considering change in deviance or the likelihood ratio test.

Finally, we compared the results with those obtained for breast cancer-specific mortality analysed using multivariate Cox regression.

We had some missing data on three of the variables included in the analyses, namely stage at diagnosis, histopathological grade, and ER status. We therefore analysed our data using two different approaches. First, we used the standard method, which is the complete case analysis (CCA), in which patients with missing data are excluded. In addition, we reanalysed our data using multiple imputation (MI), in which missing data are predicted using existing values from other variables (White *et al*, 2009). We used the same approach that we used in one of our studies (Ali *et al*, which has been accepted by the *British Journal of Cancer* ‘MD/2010/3317R’). We included all the other variables in the imputation model, in addition to the outcome of interest (overall mortality in REM models and breast-specific mortality in Cox regression models).

The results of CCA and MI were similar. We therefore reported the results of the CCA in the main manuscript and the results from the MI in the Supplementary Tables 1 and 2.

It is to be noted that, from the likelihood ratio tests, stage at diagnosis was better to be treated as a categorical variable in the REM analyses and as a continuous variable in Cox regression analyses. We reported the results of the same models, which included stage as a continuous variable, in both analyses for comparative purposes. We have shown the results of the model, which included stage as a categorical variable, in the Supplementary Table 3.

## Results

### Description of the data set

We identified 14 048 cases of invasive female breast cancer, of which 97% were confirmed histologically. Of the 14 048 patients included in our final study population, almost 40% were ⩾70 years. Table 1 summarises the clinical and tumour characteristics by age at diagnosis. There were large age-specific differences in almost all the variables of interest, with older women being more likely to be associated with poor prognostic factors. For example, 50% of patients aged 50–69 years were screen detected compared with 6% in patients aged 75–79 years, and 1% in patients aged ⩾80 years old. Older women were less likely to have had lymph nodes examined and were more likely to be diagnosed with late-stage disease. Older patients were less likely to be treated with surgery and to get local and systemic adjuvant therapies apart from adjuvant endocrine therapy, which was more commonly prescribed in older patients. In addition, older women were less likely to be treated with radiotherapy after breast-conserving surgery.

Although our data were almost complete for most of the variables, some data were missing for three of the variables included in the analysis. Missing information was much more frequent in old patients for these variables. About 30% of patients aged ⩾80 years old had data missing on at least one of the variables analysed compared with 6% in women aged 50–69 years.

Among women who died, the proportion that died of breast cancer relative to other causes declined from 70%, in women diagnosed aged 50–69 years, to 39%, in women diagnosed aged 80 and over (Table 2).

### Survival analysis

**Figure 1:**
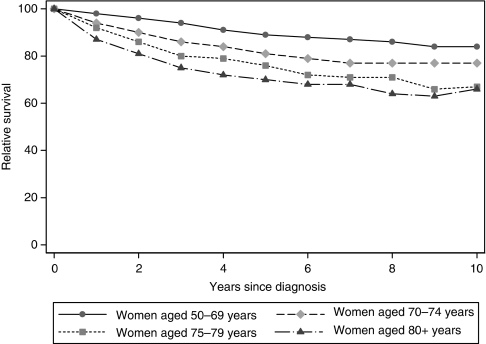
Relative survival for females diagnosed with cancer of the breast (East of England, 1999–2007).

Over a median follow-up of 4.7 years (69 834 person years), there had been 4225 deaths in the 14 048 patients. The overall 5- and 10-year RSRs were 84% (95% CI: 83–85%) and 78% (95% CI: 77–80%), respectively. As expected, given their clinical characteristics, older patients had the poorest 5- and 10-year prognosis, whereas patients diagnosed at ages 50–69 years experienced the best survival (Figure 1). RSR by other patient, tumour, and treatment characteristics are shown in Table 3.

We used REM to model the effect of age at diagnosis adjusted for other prognostic variables (Table 4). Patients ⩾70 years old had significantly higher REM in comparison with the younger age group (50–69 years) for all the models. Unadjusted REMs were 1.93 (95% CI: 1.64–2.26), 2.74 (95% CI: 2.35–3.20), and 3.88 (95% CI: 3.38–4.45) for patients aged 70–74, 75–79, and 80+ years, respectively. We then adjusted the REM for other prognostic covariates adding stage, grade, ER status, and surgery in turn (models 2–5). Each additional variable resulted in some attenuation of the age-specific REM. In the model, adjusted for all four variables, the age-specific REM estimates were substantially attenuated and the REM in the 80+ year age group was no longer statistically significant. There was little difference between the model 5 and a fully adjusted model that included stage, grade, ER status, mode of detection, deprivation quintile, hospital volume, year of diagnosis, and treatment (model 6).

The REM estimates for each variable included in the final model are shown in Table 5. It is notable that relative survival was better for patients treated in hospitals with a high volume (adjusted REM =0.69, 95% CI: 0.50–0.95). In addition, surgery was associated with the greatest increase in relative survival (REM=0.36, 95% CI: 0.30–0.44) on multivariate analysis. The hazard ratio estimates for both univariate and multivariate Cox regression using breast cancer mortality as the end point were similar to the relative excess mortality estimates. This suggests that the results of the relative survival analysis are robust.

Table 5 shows that REM declines with age in the oldest age groups, whereas breast cancer-related mortality increases with age in the Cox models. This contradiction seems to be because of missing data, because when we reanalysed our data using MI, this observation is no longer apparent (Supplementary Table 2).

## Discussion

We have used data from a population-based cancer registry to investigate age-specific breast cancer relative survival (BCRS) in the East of England. The clinical and tumour characteristics were different in older women, and this was reflected in large differences in relative survival and breast cancer-specific survival with survival being poorer in older patients. These differences were substantially reduced in a multivariate model. Some of the variables in this model reflect differences in the care pathway between older and younger patients, and other differences reflect variations in underlying tumour biology. This suggests that it might be possible to improve the prognosis in older patients by improving the manner in which breast cancer is diagnosed and treated in this age group. The results of this analysis are generally similar to those of other studies (Diab *et al*, 2000; Grosclaude *et al*, 2001; Eaker *et al*, 2006; Schonberg *et al*, 2010). The presence of residual differences in age-specific relative survival might be explained by either biological differences and/or suboptimal management (Lavelle *et al*, 2007; Schonberg *et al*, 2010). For example, it has been shown that, increasing age is an independent risk factor for nonreceipt of effective therapies after allowing for differences in tumour characteristics (Enger *et al*, 2006; Lavelle *et al*, 2007).

We conducted two complementary survival analyses, relative survival and breast cancer-specific survival. The similarity of the results suggests that relative survival is a valid method for evaluating survival time data. Its major advantages are that information on cause of death is not required and that it provides a measure of the excess mortality experienced by patients diagnosed with cancer, irrespective of whether the excess mortality is directly or indirectly attributable to the cancer (Dickman *et al*, 2004). In addition, relative survival estimates the net survival, as, by definition, it takes into account background mortality in the general population of the same age, geographical area, and time period.

We have confirmed the prognostic importance of stage at diagnosis, tumour grade, and ER status, as well as the benefit of surgery. Adjusting for stage had the biggest effect on age-specific relative survival (models 1 and 2 in Table 4). The reasons for older women presenting with late-stage disease are complex, but it is partly due to the fact that eligibility for the NHS Breast Screening Programme was restricted to women aged 50 to 69 years. Earlier diagnosis in older women has the potential to improve prognosis. In addition, there is good evidence that the higher proportion of older women presenting with more advanced stages of breast cancer is because of delay in seeking help (Ramirez *et al*, 1999).

These data also provide some evidence for suboptimal diagnostic work up in older women who were less likely to have had tumour ER status evaluated and were less likely to have had axillary lymph nodes examined. This might have resulted in inappropriate use of adjuvant hormone replacement therapy or suboptimal use of adjuvant chemotherapy. Further analysis of the implications of missing data on age-specific survival may shed more light on this issue. However, older persons are heterogeneous with respect to functional reserve, comorbidity, and personal preferences, all of which need to be considered in the treatment decision-making process. In some cases, less intensive diagnostic work up and treatment may have been appropriate. However, comorbidity data were not available to evaluate its importance in the care pathway decision making. Nevertheless, some studies have found non-rational differences in treatment among older women even after controlling for comorbidity (Giordano *et al*, 2005; Enger *et al*, 2006).

We found that women from more deprived areas have a poorer relative survival as has previously been observed by (Coleman *et al*, 2001). Unadjusted REM was 57% higher in the patients from the most deprived areas compared with those from the most affluent areas. Even after adjustment for confounders the excess was 36%. This raises an important issue in health policy regarding socioeconomic inequalities in management of patients with breast cancer.

Some researchers have argued that we do not need more research to document what we already know – that older women get suboptimal care (Mandelblatt, 2006). Instead, they stressed the need for better understanding of the biology of cancer in this population. However, our results, in concordance with others (Diab *et al*, 2000), emphasise the lack of knowledge about how best to manage older patients with breast cancer, and support the view that the benefit of therapy in older women on the natural history of the disease and the quality of life require evaluation in RCTs.

The strength of this study is its population-based case ascertainment through cancer registry, minimising the potential for selection bias as observed for hospital-based survival data and for clinical studies. The results of our study are representative of the whole population of the eastern region of England. Incomplete registration or coding mistakes, which can bias the survival estimates because of patient selection, is not a likely source of bias in this study. Cases known to registries through death certificates only (DCO) were excluded from the analysis. The proportion of microscopically verified cases in this study is 97%. Eastern Cancer Registration and Information Centre uses active follow-up, so potential bias due to incomplete follow-up is less likely. Avoidance of selection bias is the main reason to use a cancer registry-based study, however, one form of selection bias may affect these survival analyses if there are differences in assignment of patients to treatment (Grosclaude *et al*, 2001). Selection of therapy depends not only on the stage of the disease, but also on grade, endocrine receptor status, general health, patient preferences, and so on. These factors may have resulted in selection bias in this study.

Although missing data are of concern in the cancer registry data, missing data are not likely to be a problem in our analysis because we had a small proportion of cases that had missing information on few variables. In addition, when we compared the results of the CCA with the results from MI, the results were fundamentally very similar.

In conclusion, we have shown that older women with breast cancer have unfavourable clinical characteristics at presentation and poorer relative survival. Some of the survival difference can be explained by differences in the clinical characteristics by age, including stage at presentation, tumour grade, and ER status, but residual unexplained differences remain. This study has also highlighted the importance of surgery, which also accounted for some of the age-specific differences in survival. As a result, all older patients should be considered for surgery if fit enough with or without adjuvant radiotherapy and hormone therapy. Further studies are also needed to ensure the inclusion of detailed information on treatment and other factors such as comorbidity, patient preferences, waiting time for diagnosis, and/or treatment. Studies of currently available and potential molecular markers might provide better insights into the tumour biology of breast cancer in the elderly and reveal new opportunities for directing anticancer strategies in this age group.

## Figures and Tables

**Table 1:** Clinical and tumour characteristics by age group ((East of England, 1999–2007)

	**Age at diagnosis (in years)**				
	**50–69**	**70–74**	**75–79**	**80+**	**Total**
**Factor**	***N*** **(%)**	***N*** **(%)**	***N*** **(%)**	***N*** **(%)**	***N*** (**%)**
Number of patients	8514	1561	1487	2486	14 048
					
*Year of diagnosis*					
1999–2001	2559 (30)	538 (35)	525 (35)	823 (33)	4445 (32)
2002–2004	3047 (36)	554 (35)	484 (33)	838 (34)	4923 (35)
2005–2007	2908 (34)	469 (30)	478 (32)	825 (33)	4680 (33)
					
*Stage at diagnosis*					
I	4285 (51)	565 (37)	426 (30)	539 (26)	5815 (43)
II	3439 (41)	724 (47)	697 (50)	1018 (48)	5878 (44)
III	387 (5)	110 (7)	142 (10)	338 (16)	977 (7)
IV	288 (3)	127 (8)	139 (10)	214 (10)	768 (6)
Unstaged	115 (1)	35 (2)	83 (6)	377 (15)	610 (4)
					
*Tumour size (mm)*					
<20	4664 (60)	598 (44)	411 (34)	450 (27)	6123 (51)
20–49	2665 (35)	670 (49)	665 (55)	975 (59)	4975 (42)
50+	386 (5)	102 (7)	126 (10)	227 (14)	841 (7)
Missing	799 (9)	191 (12)	285 (19)	834 (34)	2109 (15)
					
*Lymph nodes*					
Negative	2512 (34)	434 (36)	392 (41)	355 (46)	3693 (36)
Positive	4822 (66)	756 (64)	560 (59)	409 (54)	6547 (64)
Missing	1180 (14)	371 (24)	535 (35)	1722 (69)	3808 (27)
					
*Grade*					
1	1634 (20)	205 (15)	167 (14)	265 (17)	2271 (19)
2	4058 (51)	762 (55)	677 (56)	906 (57)	6403 (53)
3	2284 (29)	430 (31)	367 (30)	409 (26)	3490 (29)
Missing	538 (6)	164 (11)	276 (19)	906 (36)	1884 (13)
					
*ER status*					
Negative	1181 (16)	182 (14)	165 (15)	202 (13)	1730 (15)
Positive	6383 (84)	1089 (86)	961 (85)	1407 (87)	9840 (85)
Missing	950 (11)	290 (19)	361 (24)	877 (35)	2478 (18)
					
*Mode of detection*					
Screening	4278 (50)	294 (19)	94 (6)	27 (1)	9355 (67)
Clinical	4236 (50)	1267 (81)	1393 (94)	2459 (99)	4693 (33)
					
*Nodes excised*					
<10	3925 (54)	637 (54)	462 (49)	415 (55)	5439 (53)
10–14	1710 (23)	291 (24)	279 (29)	183 (24)	2463 (24)
>14	1698 (23)	262 (22)	210 (22)	163 (21)	2333 (23)
Missing	1181 (14)	371 (24)	536 (36)	1725 (69)	3813 (27)
					
*Deprivation quintile*					
1 (Least deprived)	2086 (25)	343 (22)	294 (20)	416 (17)	3139 (22)
2	2424 (28)	417 (27)	391 (26)	681 (27)	3913 (28)
3	2193 (26)	418 (27)	421 (28)	711 (29)	3743 (27)
4	1308 (15)	277 (18)	260 (17)	480 (19)	2325 (17)
5 (Most deprived)	503 (6)	106 (7)	121 (8)	198 (8)	928 (7)
					
*Hospital volume*					
High	7797 (92)	1465 (94)	1387 (93)	2222 (89)	12 871 (92)
Low	717 (8)	96 (6)	100 (7)	264 (11)	1177 (8)
					
*Surgery*					
Yes	8156 (96)	1328 (85)	1093 (74)	1051 (42)	11 628 (83)
No	358 (4)	233 (15)	394 (27)	1435 (58)	2420 (17)
					
*Radiotherapy*					
Yes	6321 (74)	1001 (64)	783 (53)	650 (26)	8755 (62)
No	2193 (26)	560 (36)	704 (47)	1836 (74)	5293 (38)
					
*Chemotherapy*					
Yes	2502 (29)	104 (7)	50 (3)	26 (1)	2682 (19)
No	6012 (71)	1457 (93)	1437 (97)	2460 (99)	11 366 (81)
					
*Hormone therapy*					
Yes	5992 (70)	1211 (78)	1112 (75)	1903 (77)	10 218 (73)
No	2522 (30)	350 (22)	375 (25)	583 (23)	3830 (27)
					
*BCS*					
Yes	5543 (65)	772 (49)	515 (35)	506 (20)	7336 (52)
No	2971 (35)	789 (51)	972 (65)	1980 (80)	6712 (48)
					
*BCS+radiotherapy*					
Yes	4864 (77)	673 (67)	437 (56)	348 (54)	6322 (72)
No	1457 (23)	328 (33)	346 (44)	302 (46)	2433 (28)

Abbreviations: BCS=breast-conserving surgery; ER=oestrogen receptor.

**Table 2:** Breast cancer mortality in relation to all causes of mortality (follow-up period, 1999–2009)

	**Total deaths**	**Deaths from breast cancer**	**%**
50–69	1334	933	70
70–74	514	293	57
75–79	696	329	47
⩾80	1681	663	39
Total	4225	2218	53

**Table 3:** Relative 5- and 10-year survival of breast cancer patients by patient, tumour, and treatment characteristics (East of England, 1999–2007)

		**5 years**			**10 years**		
	**N**	**RSR (%)**	**LCL**	**UCL**	**RSR (%)**	**LCL**	**UCL**
*Age group (in years)*							
50–69	8506	89	89	90	84	82	85
70–74	1556	81	78	84	77	71	82
75–79	1482	76	72	79	67	59	74
80+	2451	70	66	74	66	55	78
							
*Period*							
1999–2001	4417	72	83	82	77	75	79
2002–2004	4912	74	84	83	NA^a^	NA	NA
2005–2007	4666	75	86	83	NA	NA	NA
							
*Stage at diagnosis*							
I	5814	99	98	99	97	95	100
II	5877	87	85	88	79	76	81
III	975	48	44	52	29	22	37
IV	764	14	11	17	5	2	9
							
*Grade*							
1	2271	99	97	100	98	94	100
2	6403	92	91	93	88	85	90
3	3490	74	72	76	67	64	70
							
*ER status*							
Negative	1730	68	66	71	66	62	69
Positive	9840	91	90	92	86	84	87
							
*Mode of detection*							
Screening	4693	97	96	97	95	93	97
Clinical	9302	78	76	79	70	68	72
							
*Deprivation*							
1 (least deprived)	3127	87	85	89	82	79	85
2	3895	85	83	86	80	77	83
3	3736	82	80	84	76	72	79
4	2313	83	80	85	78	73	82
5 (Most deprived)	924	82	78	86	69	61	77
							
*Hospital volume*							
High (465+ patients)	12 858	85	84	86	79	77	80
Low (<465 patients)	1137	78	75	81	75	70	80
							
*Surgery*							
Yes	11 628	92	91	93	88	86	89
No	2367	39	36	42	21	16	26
							
*Radiotherapy*							
Yes	8755	89	88	90	85	83	86
No	5240	75	74	77	67	63	70
							
*Chemotherapy*							
Yes	2682	76	74	78	65	62	68
No	11 313	86	85	87	82	80	84
							
*Hormone therapy*							
Yes	10 218	88	87	89	81	79	83
No	3777	74	73	76	70	68	73

Abbreviations: LCL=95% lower confidence limit; N=number of observations at the beginning of follow-up; RSR=relative survival rate; UCL=95% upper confidence limit.

a NA=not applicable as the follow-up for these periods is less than 10 years.

**Table 4:** 10-year relative excess mortality and 95% CIs for different age groups

	**50–69 years (reference)**	**70–74 years**			**75–79 years**			**80+ years**		
	**REM**	**REM**	**LCL**	**UCL**	**REM**	**LCL**	**UCL**	**REM**	**LCL**	**UCL**
Model 1	1	1.93	1.64	2.26	2.74	2.35	3.20	3.88	3.38	4.45
Model 2	1	1.37	1.18	1.60	1.62	1.39	1.89	1.92	1.67	2.21
Model 3	1	1.37	1.15	1.63	1.45	1.20	1.76	1.36	1.11	1.68
Model 4	1	1.50	1.24	1.81	1.49	1.21	1.84	1.47	1.17	1.84
Model 5	1	1.48	1.23	1.79	1.39	1.12	1.71	1.19	0.95	1.49
Model 6	1	1.49	1.22	1.82	1.36	1.09	1.70	1.23	0.97	1.58

Abbreviations: LCL=95% lower confidence limit; REM=relative excess mortality; UCL=95% upper confidence limit.

*Notes*: Model 1, unadjusted; Model 2, adjusted for stage; Model 3, adjusted for stage, grade; Model 4, adjusted for stage, grade, ER status; Model 5, adjusted for stage, grade, ER status, surgery; Model 6, adjusted for stage, grade, ER status, mode of detection, hospital volume, deprivation quintile, surgery, chemotherapy, radiotherapy, hormonal therapy, and year of diagnosis.

**Table 5:** Estimated 10-year relative excess mortality and hazard ratio (univariate and multivariate) and their 95% CIs

	**REM**						**Cox**					
	**Univariate**			**Multivariate**			**Univariate**			**Multivariate**		
**Variable**	**REM**	**LCL**	**UCL**	**HR**	**LCL**	**UCL**	**HR**	**LCL**	**UCL**	**HR**	**LCL**	**UCL**
*Age group (in years)*												
50–69	1.00	Ref		1.00	Ref		1.00	Ref		1.00	Ref	
70–74	1.93	1.64	2.26	1.49	1.22	1.82	1.88	1.65	2.15	1.47	1.22	1.77
75–79	2.74	2.35	3.20	1.36	1.09	1.70	2.45	2.16	2.78	1.50	1.24	1.82
80+	3.88	3.38	4.45	1.23	0.97	1.58	3.81	3.44	4.21	1.76	1.46	2.12
Period^a^	0.93	0.86	1.00	0.88	0.80	0.96	0.91	0.86	0.96	0.90	0.83	0.97
Stage^a^	4.57	4.32	4.84	3.12	2.85	3.41	4.05	3.87	4.23	2.95	2.73	3.18
Grade^a^	3.73	3.21	4.32	2.26	1.97	2.59	2.83	2.60	3.08	2.09	1.87	2.33
ER positive	0.26	0.23	0.30	0.60	0.50	0.73	0.34	0.31	0.38	0.55	0.47	0.66
Screen detected	0.08	0.06	0.12	0.66	0.54	0.81	0.17	0.15	0.20	0.67	0.56	0.80
Deprivation quintile^a^	1.12	1.07	1.17	1.08	1.02	1.14	1.13	1.09	1.16	1.06	1.01	1.11
High hospital volume	1.45	1.22	1.71	0.69	0.50	0.95	1.36	1.18	1.56	0.79	0.61	1.03
Surgery	0.07	0.06	0.08	0.36	0.30	0.44	0.12	0.11	0.14	0.39	0.32	0.46
Radiotherapy	0.34	0.30	0.38	0.85	0.73	0.98	0.55	0.50	0.60	1.05	0.92	1.20
Chemotherapy	1.78	1.60	1.98	1.10	0.93	1.29	1.69	1.54	1.86	1.21	1.04	1.41
Hormonotherapy	0.41	0.36	0.45	0.71	0.59	0.86	0.59	0.54	0.64	0.88	0.75	1.03

Abbreviations: CI=confidence interval; HR=Hazards ratio; LCL=95% lower confidence limit; REM=relative excess mortality; UCL=95% upper confidence limit.

a These variables were treated as continuous variables, giving hazard ratios per unit increase.
