# Supplementary material for: Patient and tumour characteristics, management, and age-specific survival in women with breast cancer in the East of England
Source: Br J Cancer. 2011 Feb 15;104(4):564–70. doi: 10.1038/bjc.2011.14 (PMC3049594; doi:10.1038/bjc.2011.14)
Supplement: Supplementary Tables 1–3 [file bjc201114x1.doc]

Supplementary table 1 10-year Relative excess mortality and 95 CIs for different age groups using multiple imputation

|  | **50-69 (ref)** | **70-74** | | | **75-79** | | | **80+** | | |
| --- | --- | --- | --- | --- | --- | --- | --- | --- | --- | --- |
|  | REM | REM | LCL | UCL | REM | LCL | UCL | REM | LCL | UCL |
| Model 1 | 1 | 1.93 | 1.64 | 2.26 | 2.74 | 2.35 | 3.20 | 3.88 | 3.38 | 4.45 |
| Model 2 | 1 | 1.39 | 1.20 | 1.61 | 1.72 | 1.49 | 1.99 | 2.01 | 1.77 | 2.29 |
| Model 3 | 1 | 1.42 | 1.23 | 1.65 | 1.69 | 1.46 | 1.95 | 2.20 | 1.93 | 2.50 |
| Model 4 | 1 | 1.49 | 1.29 | 1.73 | 1.75 | 1.51 | 2.02 | 2.34 | 2.06 | 2.66 |
| Model 5 | 1 | 1.32 | 1.14 | 1.53 | 1.48 | 1.28 | 1.70 | 1.64 | 1.44 | 1.87 |
| Model 6 | 1 | 1.26 | 1.07 | 1.47 | 1.44 | 1.23 | 1.68 | 1.54 | 1.33 | 1.79 |

Notes

Model 1: Unadjusted

Model 2: Adjusted for stage

Model 3: Adjusted for stage, grade

Model 4: Adjusted for stage, grade, ER status

Model 5: Adjusted for stage, grade, ER status, surgery

Model 6: Adjusted for stage, grade, ER status, mode of detection, hospital volume, deprivation quintile, surgery, chemotherapy, radiotherapy, hormonal therapy, and year of diagnosis

**Supplementary table 2 Estimated 10-year relative excess mortality and hazard ratio (univariate and multivariate) and their 95% CIs Using multiple imputation**

| **Variable** | **REM** | | | | | | **Cox** | | | | | |
| --- | --- | --- | --- | --- | --- | --- | --- | --- | --- | --- | --- | --- |
|  | Univariate | | | Multivariate | | | Univariate | | | Multivariate | | |
|  | REM | LCL | UCL | HR | LCL | UCL | HR | LCL | UCL | HR | LCL | UCL |
| Age group |  |  | |  |  | |  |  | |  |  | |
| 50-69 | 1.00 | Ref | | 1.00 | Ref | | 1.00 | Ref | | 1.00 | Ref | |
| 70-74 | 1.93 | 1.61 | 2.24 | 1.26 | 1.07 | 1.47 | 1.88 | 1.65 | 2.15 | 1.33 | 1.15 | 1.55 |
| 75-79 | 2.74 | 2.44 | 3.05 | 1.44 | 1.23 | 1.68 | 2.45 | 2.16 | 2.78 | 1.43 | 1.23 | 1.67 |
| 80+ | 3.88 | 3.61 | 4.15 | 1.54 | 1.33 | 1.79 | 3.81 | 3.44 | 4.21 | 1.79 | 1.54 | 2.08 |
| Period* | 0.93 | 0.78 | 1.07 | 0.89 | 0.84 | 0.95 | 0.91 | 0.86 | 0.96 | 0.90 | 0.84 | 0.95 |
| Stage* | 4.31 | 4.08 | 4.55 | 2.67 | 2.50 | 2.85 | 3.96 | 3.79 | 4.14 | 2.73 | 2.58 | 2.90 |
| Grade* | 2.72 | 2.46 | 3.01 | 1.74 | 1.59 | 1.91 | 2.49 | 2.30 | 2.69 | 1.84 | 1.68 | 2.03 |
| ER positive | 0.30 | 0.27 | 0.33 | 0.65 | 0.57 | 0.75 | 0.36 | 0.32 | 0.40 | 0.59 | 0.50 | 0.69 |
| Screen detected | 0.08 | 0.06 | 0.12 | 0.60 | 0.50 | 0.72 | 0.17 | 0.15 | 0.20 | 0.61 | 0.52 | 0.72 |
| Deprivation quintile* | 1.12 | 1.03 | 1.21 | 0.96 | 0.82 | 1.12 | 1.13 | 1.09 | 1.16 | 1.06 | 1.03 | 1.11 |
| High hospital volume | 1.45 | 1.12 | 1.78 | 1.07 | 1.02 | 1.11 | 1.36 | 1.18 | 1.56 | 1.10 | 0.93 | 1.29 |
| Surgery | 0.07 | 0.06 | 0.08 | 0.31 | 0.27 | 0.35 | 0.12 | 0.11 | 0.14 | 0.37 | 0.32 | 0.42 |
| Radiotherapy | 0.34 | 0.11 | 0.56 | 0.77 | 0.69 | 0.86 | 0.55 | 0.50 | 0.60 | 0.99 | 0.89 | 1.09 |
| Chemotherapy | 1.78 | 1.57 | 1.99 | 0.77 | 0.68 | 0.88 | 1.69 | 1.54 | 1.86 | 1.11 | 0.98 | 1.26 |
| Hormonotherapy | 0.41 | 0.19 | 0.62 | 0.56 | 0.49 | 0.63 | 0.59 | 0.54 | 0.64 | 0.77 | 0.67 | 0.87 |

NB: * These variables were treated as continuous variables, giving hazard ratios per unit increase.

NB: In REM models, end of follow-up was included in this model (10 periods, 1-year each)

**Supplementary table 3 Estimated 10-year relative excess mortality (univariate and multivariate) and their 95% CIs (where is stage was treated as categorical variable)**

| **Variable** | **REM** | | | | | |
| --- | --- | --- | --- | --- | --- | --- |
|  | Univariate | | | Multivariate | | |
|  | REM | LCL | UCL | HR | LCL | UCL |
| Age group |  |  | |  |  | |
| 50-69 | 1.00 | Ref | | 1.00 | Ref | |
| 70-74 | 1.93 | 1.64 | 2.26 | 1.49 | 1.22 | 1.82 |
| 75-79 | 2.74 | 2.35 | 3.20 | 1.36 | 1.09 | 1.70 |
| 80+ | 3.88 | 3.38 | 4.45 | 1.23 | 0.97 | 1.58 |
| Period* | 0.93 | 0.86 | 1.00 | 0.88 | 0.80 | 0.96 |
| Stage |  |  |  |  |  |  |
| I | 1.00 | Ref |  | 1.00 | Ref |  |
| II | 15.30 | 5.73 | 40.86 | 3.39 | 2.54 | 4.53 |
| III | 81.82 | 30.63 | 218.56 | 11.96 | 8.71 | 16.42 |
| IV | 287.19 | 107.91 | 764.33 | 30.05 | 21.50 | 41.99 |
| Missing | 90.78 | 33.76 | 244.12 | 7.90 | 4.74 | 13.17 |
| Grade* | 3.73 | 3.21 | 4.32 | 2.26 | 1.97 | 2.59 |
| ER positive | 0.26 | 0.23 | 0.30 | 0.60 | 0.50 | 0.73 |
| Screen detected | 0.08 | 0.06 | 0.12 | 0.66 | 0.54 | 0.81 |
| Deprivation quintile* | 1.12 | 1.07 | 1.17 | 1.08 | 1.02 | 1.14 |
| High hospital volume | 1.45 | 1.22 | 1.71 | 0.69 | 0.50 | 0.95 |
| Surgery | 0.07 | 0.06 | 0.08 | 0.36 | 0.30 | 0.44 |
| Radiotherapy | 0.34 | 0.30 | 0.38 | 0.85 | 0.73 | 0.98 |
| Chemotherapy | 1.78 | 1.60 | 1.98 | 1.10 | 0.93 | 1.29 |
| Hormonotherapy | 0.41 | 0.36 | 0.45 | 0.71 | 0.59 | 0.86 |
